# Supplementary material for: Integrative analysis of single-cell expression data reveals distinct regulatory states in bidirectional promoters
Source: Epigenetics Chromatin. 2018 Nov 10;11:66. doi: 10.1186/s13072-018-0236-7 (PMC6230222; doi:10.1186/s13072-018-0236-7)
Supplement: Supplementary file 1 — Additional file 1. Supplementary methods and results. [file 13072_2018_236_MOESM1_ESM.pdf]

Integrative analysis of single cell expression data reveals distinct regulatory states in bidirectional promoters

Supplemental Methods

GRO-cap and CAGE expression estimation

GRO-cap data for K562 cell was downloaded from GEO under accession GSE60456 provided by Core et al. [2014] and CAGE data for both K562 and HepG2 samples from ENCODEConsortium [2012]. The count of the reads overlapping with a window in the region [0,+100] bps downstream of a gene's TSS is used to define GRO-cap or CAGE derived expression.

Bulk RNA expression quantification

BAM files of RNA-seq reads for HepG2 were produced with TopHat 2.0.11 [Kim et al., 2013], with Bowtie 2.2.1 [Langmead and Salzberg, 2012] and NCBI build 37.1 with parameters: *–library-type fr-firststrand* and *–b2-very-sensitive*. Cufflinks was used for gene expression computation [Trapnell et al., 2012] using GENCODE release 19 (GRCh37.p13).

Small RNA abundance in BPs

The BAM alignment files for small RNA data measured at the nuclear fraction of the HEpG2 and K562 cells were obtained from ENCODEConsortium [2012]. Then, bamCoverage from bedtools was applied to generate the bedgraph files, which then the binning approach explained in the previous section was performed to achieve the small RNA profile defined around the BPs. For illustration purposes, all values larger than 200 were set to 200.

Mapping of ChIP-seq data

Reads were mapped to the 1000 genomes phase 2 assembly of the human reference genome (NCBI build 37.1, downloaded from) with a hardware-accelerated implementation of Burrows-Wheeler Aligner BWA aln version 0.6.2 [Liu et al., 2012] with -q 20, and BWA 0.6.2 sampe with -a 1000.

Merging and duplicate marking was performed with Picard version 1.125 (`http://broadinstitute.github.io/picard`).

Measuring H3K36me3 in *transcripts span* of BPs

The H3K36me3 ChIP-seq reads are counted in the region starting from the TSS of a bidirectional gene extending down to the *transcripts span* partitioned into 10 bins. It is worth noting that the bin sizes might vary between genes as they have variable *transcripts span* lengths. Therefore, read counts are normalized according to the bin size.

Measuring average methylation in BPs

WGBS-seq data for HepG2 was produced by DEEP and for K562 was obtained from ENCODEConsortium [2012]. Both files were processed using the RnBeads package in R [Assenov et al., 2014] to measure the average methylation levels around the TSSs. Briefly, for each TSS, the methylation level was computed in a 2 kb window (partitioned into bins of 100 bp) downstream of the L and the H gene, respectively (in total 40 bins). Additionally, the methylation level was measured within the region between the TSSs of L and H genes. Finally, the results were concatenated in genomic order (in total a vector of size 41).

Measuring G-C content in BPs

GC-content profiles were computed based on the human GRCh37 reference genome. For each TSS GC-content was computed in a 2 kb window (partitioned into bins of 100 bp) downstream of the L and the H gene, respectively (in total 40 bins). Additionally, the GC-content was measured within the region between the TSSs of L and H genes. For visualization the results were concatenated in genomic order (in total a vector of size 41).

Measuring 3'UTR length in BPs

3'UTR coordinates for our BPs were retrieved from annotated ENSEMBL genes (GRCh37.75) to show the 3'UTR length of the highly and lowly expressed genes, particularly, in the stable and unstable categories as illustrated in Supplementary Figure 4C. The Mann-Whitney test was used between the highly and lowly expressed genes within each category to compute the p-values with the 0.05 cutoff for significance calling.

Chromatin state segmentation score

We acquired the 18-states ChromHMM [Ernst and Kellis, 2012] annotation for both cell lines, for HepG2 produced by DEEP, and for K562 downloaded

from Roadmap [Consortium et al., 2015]. For simplicity, we collapsed all TSS related states to one state called, TSS. Similarly, we defined Enhancer and Repressed states and assigned all the remaining states to Others, yielding four summarized states in general. Later, for each gene g we defined a window, W_g, starting at the TSS of the gene and extending up to the size of the *transcripts span*, see above. We then computed the average number of bases having a particular chromatin state, s, overlapping in that window. We called this value $ChromScore_g^s$, described as follows:

$$ChromScore_g^s = \frac{\Sigma\{|R| : R \subseteq W_g \ and \ state(R) = s\}}{W_g}, \tag{1}$$

where R defines a region in the genome, $|R|$ designates the size of this region, and $state(R)$ denotes the chromatin state assigned by ChromHMM to region R. It should be noted that since the ChromHMM state annotation is continuous across the genome, the following equation holds:

$$\Sigma_{s \in \{TSS, Enhancer, Repressed, Others\}} ChromScore_g^s = 1\,, \tag{2}$$

and thus $ChromScore$ is properly normalized to account for a difference in *transcripts span* per gene. To assign ChromScore to a cluster of genes, C, (defining the four transcription states introduced earlier), we formulated the following:

$$ChromScore_C^s = \Sigma_{g \in C} ChromScore_g^s, \tag{3}$$

Later, as the last step, we convert the $ChromScore_C^s$ into percentages to make the score comparable across different clusters of genes with different gene sizes:

$$percent(ChromScore_C^s) = \frac{ChromScore_C^s}{\Sigma_{s \in \{TSS, Enhancer, Repressed, Others\}} ChromScore_C^s}\,. \tag{4}$$

References

Y. Assenov, F. Müller, P. Lutsik, J. Walter, T. Lengauer, and C. Bock. Comprehensive analysis of DNA methylation data with RnBeads. *Nat Meth*, 11(11):1138–1140, 11 2014. URL http://dx.doi.org/10.1038/nmeth.3115.

R. E. Consortium, A. Kundaje, W. Meuleman, J. Ernst, M. Bilenky, A. Yen, A. H. Moussavi, P. Kheradpour, Z. Zhang, J. Wang, M. J. Ziller, V. Amin, J. W. Whitaker, M. D. Schultz, L. D. Ward, A. Sarkar, G. Quon, R. S. Sandstrom, M. L. Eaton, Y. Wu, A. R. Pfenning, X. Wang, M. Claussnitzer, Y. Liu, C. Coarfa, R. A. Harris, N. Shoresh, C. B. Epstein, E. Gjoneska, D. Leung, W. Xie, R. D. Hawkins, R. Lister, C. Hong,

P. Gascard, A. J. Mungall, R. A. Moore, E. Chuah, A. Tam, T. K. Canfield, R. S. Hansen, R. Kaul, P. J. Sabo, M. S. Bansal, A. Carles, J. R. Dixon, K. Farh, S. Feizi, R. Karlic, A. Kim, A. Kulkarni, D. Li, R. F. Lowdon, G. Elliott, T. R. Mercer, S. J. Neph, V. Onuchic, P. Polak, N. Rajagopal, P. Ray, R. C. Sallari, K. T. Siebenthall, N. A. Sinnott-Armstrong, M. Stevens, R. E. Thurman, J. Wu, B. Zhang, X. Zhou, A. E. Beaudet, L. A. Boyer, P. L. D. Jager, P. J. Farnham, S. J. Fisher, D. Haussler, S. J. M. Jones, W. Li, M. A. Marra, M. T. McManus, S. R. Sunyaev, J. A. Thomson, T. D. Tlsty, L. Tsai, W. Wang, R. A. Waterland, M. Q. Zhang, L. H. Chadwick, B. E. Bernstein, J. F. Costello, J. R. Ecker, M. Hirst, A. Meissner, A. Milosavljevic, B. Ren, J. A. Stamatoyannopoulos, T. Wang, and M. Kellis. Integrative analysis of 111 reference human epigenomes open. *Nature*, 518(7539):317–330, 2015. doi: 10.1038/nature14248. URL `https://doi.org/10.1038/nature14248`.

L. J. Core, A. L. Martins, C. G. Danko, C. T. Waters, A. Siepel, and J. T. Lis. Analysis of nascent RNA identifies a unified architecture of initiation regions at mammalian promoters and enhancers. *Nature Genetics*, 46(12): 1311–1320, nov 2014. ISSN 1061-4036.

ENCODEConsortium. An integrated encyclopedia of DNA elements in the human genome. *Nature*, 489(7414):57–74, 09 2012.

J. Ernst and M. Kellis. ChromHMM: automating chromatin-state discovery and characterization. *Nature Methods*, 9(3), 2012. doi: 10.1093/nar/gkv1495.

D. Kim, G. Pertea, C. Trapnell, H. Pimentel, R. Kelley, and S. Salzberg. Tophat2: accurate alignment of transcriptomes in the presence of insertions, deletions and gene fusions. *Genome Biology*, 14(4):R36, 2013. ISSN 1465-6906. doi: 10.1186/gb-2013-14-4-r36.

B. Langmead and S. L. Salzberg. Fast gapped-read alignment with bowtie 2. *Nat Meth*, 9(4):357–359, 04 2012.

Y. Liu, K. D. Siegmund, P. W. Laird, and B. P. Berman. Bis-SNP: combined DNA methylation and SNP calling for Bisulfite-seq data. *Genome biology*, 13(7):R61, jan 2012. ISSN 1465-6914.

C. Trapnell, A. Roberts, L. Goff, G. Pertea, D. Kim, D. R. Kelley, H. Pimentel, S. L. Salzberg, J. L. Rinn, and L. Pachter. Differential gene and transcript expression analysis of RNA-seq experiments with TopHat and Cufflinks. *Nat. Protocols*, 7(3):562–578, 03 2012.

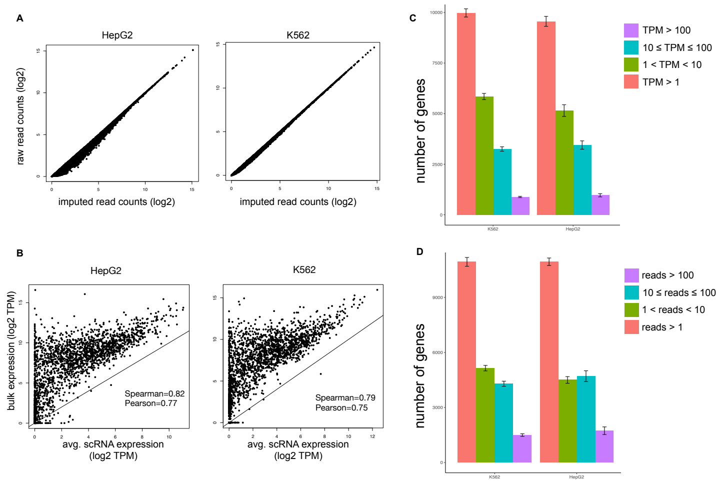

Figure S1: A) Comparison between raw and imputed read counts for both HepG2 and K562 cell lines. B) Comparison of average single cell RNA-seq expression (x-axis) versus bulk RNA-seq expression (y-axis) in bidirectional genes for HepG2 and K562 cell lines. C) Number of expressed genes according to the four intervals defined on TPM values per cell. $TPM > 1$ shows the overall number of genes detected to have expression larger than 1 TPM. On average, there are over 7,000 genes expressed for both cell lines. D) Similar to (C) but for read counts.

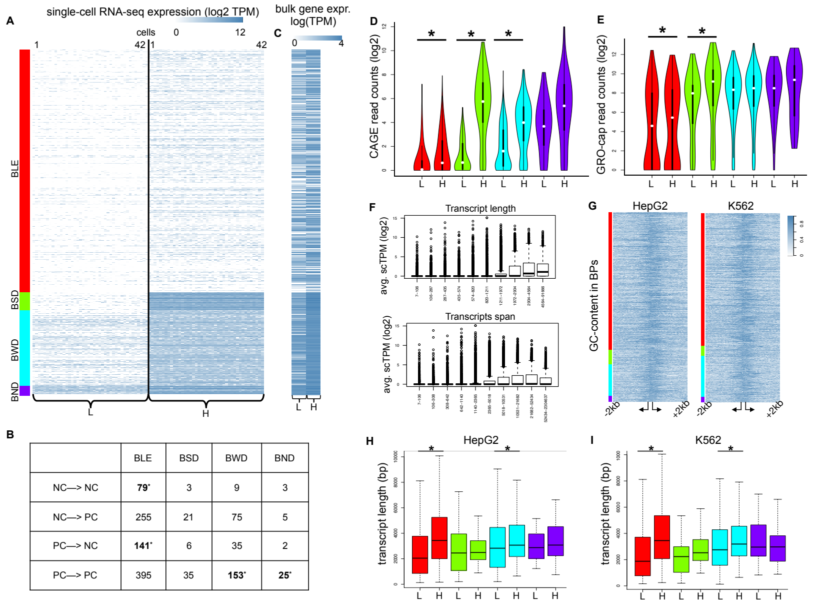

	BLE	BSD	BWD	BND
NC—> NC	**79***	3	9	3
NC—> PC	255	21	75	5
PC—> NC	**141***	6	35	2
PC—> PC	395	35	**153***	**25***

Figure S2: Single cell RNA-seq expression in bidirectional promoters. A) Hierarchical clustering of the K562 single cell transcript expression matrix visualized as a heatmap (log2, TPM) and grouped into four distinct clusters (*BLE*, *BSD*, *BWD*, *BND*). B) Number of BPs falling into the gene product categories (NC→NC, NC→PC, etc.). Statistically enriched values are shown in bold (Hypergeometric test p<0.05). C) Heatmap of bulk RNA-seq expression in K562 cells (log2, TPM), arranged according to A. D) CAGE read counts, measured for each bidirectional gene (L and H), shown for each transcription state. Color code as in A. Significant differences are marked with * (paired and two-sided Mann-Whitney test, $p < 0.05$). E) The same as D except that the result is shown for GRO-cap read counts. F) Effects of transcript length and *transcripts span* on average single cell TPM expression in all genes. G) Heatmaps of the G-C content measured around the TSSs of bidirectional genes (see Materials and Methods) for HepG2 (left panel, color code as in Figure 1D) and K562 (right panel, color code as in A) samples. H) Distributions of transcript length of L and H genes in HepG2 (color code as in Figure 1D) and I) K562 (color code as in A). Significant differences are marked by * (Mann-Whitney paired and two-sided test, p-value < 0.05).

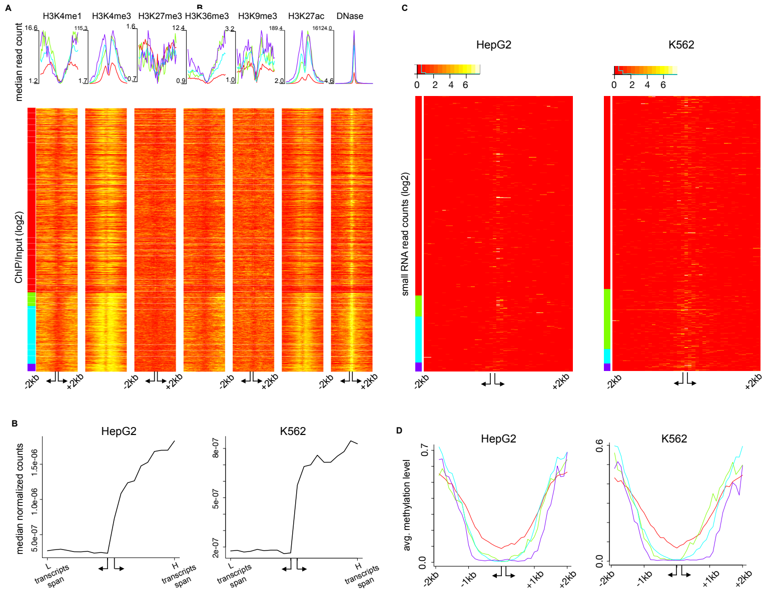

Figure S3: Epigenetic characteristics in transcription states. A) Histone modification (ChIP/Input) and DNA-seq1 (raw read counts) shown as median profiles (top panel) and log-transformed values as heatmap (bottom panel). Arrangement of genes as in Figure S2A. The reads are measured in 40 bins of size 100 bp forming a window of size 4000 bp centered around the TSSs, with an additional variable bin between the TSSs. B) Measured H3K36me3 ChIP-seq counts in bins of variable size covering the region starting from the TSS of L and H genes extending down to the*transcripts span*. C) Small RNA abundance heatmaps measured similar to A for HepG2 (left panel) and K562 (right panel). For better visibility, bins with more than 200 reads were set to 200. D) Average methylation profiles measured by WGBS-seq read counts in bins of 100 bp following the approach explained in A, shown for both HepG2 (left panel) and K562 (right panel).

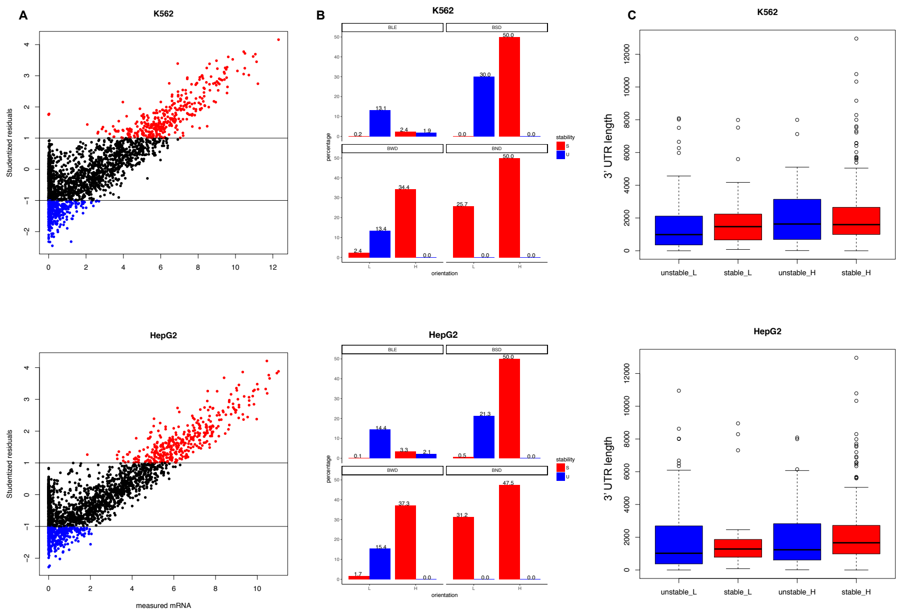

Figure S4: Prediction of RNA stability. A) Scatter plot showing the studentized residuals versus the measured mRNA as average single cell transcript expression for both HepG2 and K562 samples. B) Percentage of L or H genes inferred as stable or unstable per state. C) The 3'UTR length distribution shown for L or H genes per each stability category.

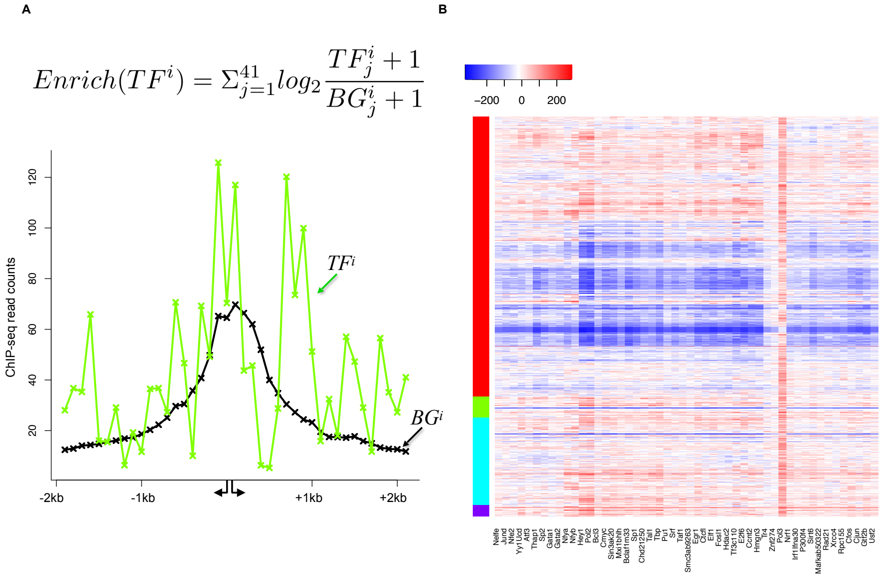

Figure S5: Transcription factor enrichment. A) Enrichment score, $Enrich(TF^i)$, formula to compute the enrichment of TFs tailored for BPs. At the bottom, the binding profile of TF^i for a BP is shown. The curve shown in black represents the background defined based on the bin-wise median of TF^i binding across all BPs. The example demonstrates the effectiveness of the $Enrich(TF^i)$ score in capturing the spatial differences between true TF signal and the background. B) Heatmap of 50 TF enrichment scores (log ratio against background) for each BP (row) in K562 cells.

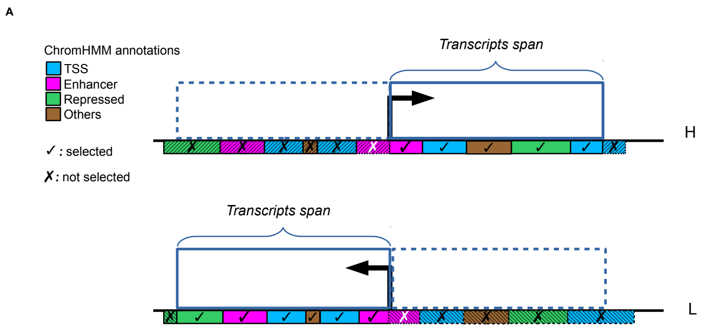

Figure S6: A schematic representation of computing the segmented genomic regions using ChromHMM for a region defined at the *transcripts span* of either genes (L and H). The overlapping regions are taken into account for computing the *ChromScore* (see Methods).
